# Antiosteoporotic effect of orally administered yolk-derived peptides on bone mass in women

**DOI:** 10.1002/fsn3.94

**Published:** 2014-02-28

**Authors:** Adham M Abdou, Kazuya Watabe, Tetsuro Yamane, Tadayuki Isono, Yoshitaka Okamura, Seiji Kawahito, Kazuhito Takeshima, Kazuyuki Masuda, Mujo Kim

**Affiliations:** 1 Pharma Foods International Co., Ltd.Kyoto, 615-8245, Japan; 2 Matsushita Memorial HospitalMoriguchi City, Osaka, 570-8540, Japan; 3 Wada Calcium Pharmaceutical Co., Ltd.Osaka, 538-0043, Japan; 4 Iwaki & Co., Ltd.Tokyo, 103-8403, Japan

**Keywords:** Antiosteoporotic, bone density, bone markers, osteoporosis, women, yolk-derived peptides

## Abstract

The main objective of this study was to verify the effect of oral intake of a yolk-derived peptide preparation (HYP) obtained by enzymatic hydrolysis of yolk water-soluble protein on bone markers and bone density in 65 perimenopausal women with an average age of 47.6 ± 5.2 years. Subjects were divided into three groups, and then enrolled in a 6-month, randomized, double-blind clinical trial. Bone formation and resorption markers were measured at 0, 3, and 6 months, while bone mineral density (BMD) in the lumbar spines was measured at 0 and 6 months. Although the bone formation marker levels showed the similarity changes among the groups, bone resorption markers in the test HYP group were significantly decreased after 3 and 6 months in comparison to other groups (*P* < 0.05). After 6 months, BMD in the test HYP group maintained at healthy numerical values whereas BMD values were decreased in other groups. Hence HYP would be an antiosteoporotic agent originated from natural food to maintain bone health, especially for women.

## Introduction

Many factors associated with the variability in total bone mass and bone mineral density (BMD) include hereditary, hormonal status, diet, exercise, and lifestyle. Bone is continuously formed and resorbed throughout the human life cycle, and a reduction in mass results from bone resorption exceeding bone formation (Faine 1997; Katagiri and Takahashi 2002). Progressive and generalized bone loss occurs in women following menopause, frequently leading to osteoporosis, which is defined as low bone mass and microarchitectural deterioration leading to increase in skeletal fragility and high risk of fractures (Feng et al. 2009; Napoli et al. 2011).

Although development of more effective treatment strategies continues to investigate new agents for treatment of osteoporosis, none of them is able to completely prevent osteoporotic fractures. All present agents seem to improve the quality of life of osteoporotic patients insufficiently due to their general efficacy, mode of application, or various side effects. Thus, the urge for more effective and safe antiosteoporotic agents remains high (Miyamoto et al. 1997; Yasuda et al. 2005).

Egg is broadly recognized as a very valuable source of proteins for human nutrition and is known to contain many substances with biological functions beyond basic nutrition. The advances in protein separation help in exploring the biological functions of egg proteins. The biologically active egg proteins would provide nutritional and functional ingredients that enhance human health (Anton et al. 2006). The new advances in protein bioengineering help to explore numerous potentials for releasing biologically functional peptides due to degradation of egg proteins by specific enzymes. The resultant peptides could show enhanced or biologically new function with improved stability and/or solubility. This aspect has been studied and some peptides, which exhibit various activities such as antihypertensive, bone growth-promoting, anticancer, or exaggerated antimicrobial activities, have been found (Anton et al. 2006; Abdou et al. 2013).

Some investigations were carried out to explore the biologically active substances in hen egg that would initiate and enhance bone growth. It has been reported that egg yolk phosvitin and their peptides enhanced Ca-binding capacity and significantly increased Ca incorporation into bones (Jiang and Mine 2001; Choi et al. 2005). Moreover, in our previous studies, it has been found that a specific yolk water-soluble protein fraction (YP) has bone growth promotion activity both in vitro and in vivo (Leem et al. 2004; Kim et al. 2008; Abdou et al. 2013). These findings encouraged us to find out the functional peptides that would have bone growth promotion activity. Different enzymes were used to hydrolyze YP and the antiosteoporotic effect of peptide preparations was investigated. A novel yolk-derived peptide preparation (HYP) showed a remarkable activation of the preosteoblastic MC3T3-E1 cell proliferation and it potently suppressed osteoclastogenesis. Moreover, ovariectomized rats fed on HYP showed an osteoprotective effect through increasing the BMD and prevented the cancellous bone loss induced by ovariectomy (Kim et al. 2011).

So, to extend the work, the purpose of this study was to verify the effect of HYP on bone markers and BMD in perimenopausal women in a 6-months, double-blind, randomized clinical trial.

## Materials and Methods

### Determination of yolk-derived peptide preparation characteristics

The ingredient (HYP) was provided by Pharma Foods International Co., Ltd. (Kyoto, Japan). It was prepared from egg yolk water-soluble protein (YP) that had undergone hydrolysis with alkaline protease at 60°C for 3 h. The molecular weight distribution of HYP was analyzed by high-performance liquid chromatography (HPLC) equipped with YMC-Pack Diol-60 column (500 × 8 mm I.D.; YMC, Kyoto, Japan). The mobile phase was 0.1 mol/L KH_2_PO_4_–K_2_HPO_4_ containing 0.2 mol/L NaCl (pH 7.0)/acetonitrile (70/30, v/v) at 0.7 mL/min. Absorbance was measured at 215 nm.

### Subjects

Sixty-five women were enrolled in a randomized, double-blind, clinical study for 6 months. The mean age of participants was 47.6 ± 5.2 years, ranging from 44 to 59 years. Subjects must have been in generally good health, with no diseases known to contribute to osteoporosis, not have been treated or taking medications for osteoporosis. Considering ethical issues in asking whether the participants had reached menopause, the test was conducted without identifying the numbers of postmenopausal women. All participants provided written informed consent. The study protocol was approved by the ethics committee of Matsushita Memorial Hospital, Osaka, Japan.

### Protocol

Three different granulated powder formulations were prepared by Wada Calcium Pharmaceutical Co., Ltd (Osaka, Japan). The composition of the powder formulations (WA-1, WA-2, and WA-3) is illustrated in Table 1. The subjects were divided randomly into three groups; 21 subjects (WA-1), 21 subjects (WA-2), and 23 subjects (WA-3) receiving their corresponding formula; respectively. The daily intake of HYP in the test WA-3 group was set as 100 mg per subject, calculated to apply its ED50, obtained from previous animal study (Leem et al. 2004), to “Conversion of Animal Dose to Human Equivalent Dose (HED) Based on Body Surface Area” issued by U.S. Department of Health and Human Services, Food and Drug Administration, Center for Drug Evaluation and Research (CDER) (2005). Subjects were administered the powder formulations twice a day (morning and evening) for 6 months consecutively. All powder formulations were similar in appearance and taste.

**Table 1:** Composition of powder formulations received by subjects in each group

Ingredients	WA-1 (content/dose)	WA-2 (content/dose)	WA-3 (content/dose)
Calcium	300 mg	300 mg	300 mg
Magnesium	175 mg	175 mg	175 mg
Vitamin K2	–	25 *μ*g	25 *μ*g
Vitamin D3	–	2.5 *μ*g	2.5 *μ*g
Collagen	–	150 mg	150 mg
Isoflavone	–	10 mg	10 mg
HYP	–	–	50 mg

HYP, yolk-derived peptide preparation.

### Biochemical examination

Blood and urine analysis have been conducted at 0, 3, and 6 months on fasting blood and urine samples obtained from subjects in the morning between 0900 and 1200 h. Blood and serum samples were analyzed using glutamate-oxaloacetate (GOT), glutamate-pyruvate transaminase (GPT), alkaline phosphatase (ALP), lactate dehydrogenase (LDH), creatine phosphokinase (CPK), total bilirubin (T-Bil), total protein (TP), albumin (ALB), albumin/globulin ratio, total cholesterol (Chl), blood urea nitrogen (BUN), uric acid (UA), creatinine (CRE), C-reactive protein, glucose, white blood cell count (WBC), platelet count (PLT), red blood cell count (RBC), hemoglobin (Hb), hematocrit (Ht), mean corpuscular volume (MCV), mean corpuscular hemoglobin (MCH), mean corpuscular hemoglobin concentration (MCHC), neutrophil, eosinophil granulocyte, basophil, lymphocyte, and monocyte. Urine samples were analyzed for protein and sugar in urine.

### Bone marker examination

Specimens were obtained from fasting morning blood or urine. All samples from an individual subject were measured in the same assay run. The examinations for bone formation and bone resorption markers were carried out at 0, 3, and 6 months using commercially available kits according to the manufacturer's instructions. For bone formation markers; osteocalcin (OCN) was measured using immunoradiometric (IRMA) assay kits (Mitsubishi chemical Medience Corporation, Tokyo, Japan), while bone-specific alkaline phosphatase (BAP) was measured using the chemiluminescent enzyme immunoassay (CLEIA) kits (Beckman Coulter Inc., Tokyo, Japan). For bone resorption markers, tartarate-resistant acid phosphatase (TRAP) was determined using enzyme immnunoassay kits (DS Pharma Biomedical Co., Ltd, Osaka, Japan), while N-terminal collagen telopeptide (NTx) was determined using the enzyme-linked immunosorbent assay (ELISA) kits (Alere Medical Co., Ltd, Tokyo, Japan).

### Bone mineral density

BMD of the lumbar spines was measured at baseline and 6 months by dual energy X-ray absorptiometry (DEXA). The same DEXA was used for all subjects and for serial measurements.

### Statistical analysis

Analysis was performed for 65 subjects who completed the 6 months study. Two subjects were dropped out before the first inspection. All statistical analysis was conducted with SPSS statistical software package ver. 11.1J (SPSS Japan Inc., Tokyo, Japan). Statistical comparisons were made by using one-way analysis of variance (ANOVA) and Fisher's PLSD method. Data are presented as means ± SEM.

## Results and Discussion

### Yolk-derived peptide preparation characteristics

**Figure 1:**
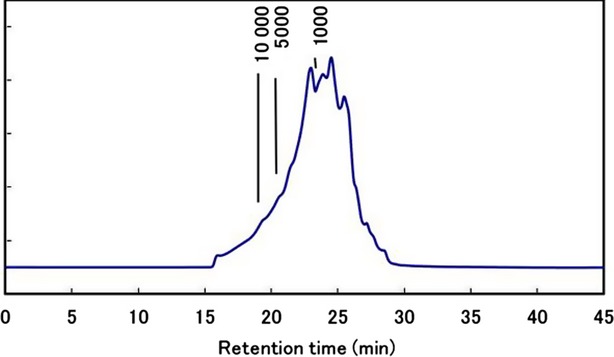
Analysis of molecular weight distributions of yolk-derived peptide preparation.

The molecular weight distribution analysis of HYP is shown in Figure 1. Chromatography showed that about 85% of the HYP had a molecular weight of less than 3000 Da and about 70% less than 1000 Da. This relatively low molecular weight of HYP would enhance the absorption and bioavailability of the functional peptides in human (Feng et al. 2009). Effect of different pH, temperatures, and different digestive enzymes against HYP in vitro has been examined and showed its stability for heat up to 140°C, pH 2.0–9.0, and stability to enzymatic digestion (data not shown), therefore, the oral intake of HYP would not undergo further degradation in subjects in this study.

### Biochemical examination

No differences were observed among three groups in all subjects. All scales for blood and urine analysis were within the normal range and no effects on health were confirmed (data not shown).

### Bone formation markers

**Figure 2:**
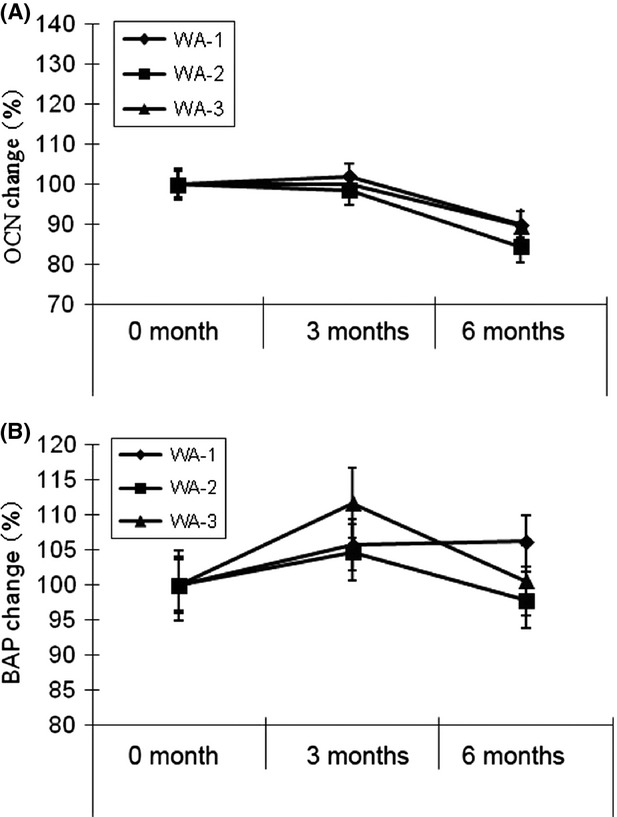
Osteocalcin (OCN) change rate (A) and bone-specific alkaline phosphatase (BAP) change rate (B) in women (mean ± SEM) in WA-1, WA-2, and WA-3 groups after 0, 3, and 6 months.

For analysis of bone formation markers levels, as shown in Figure 2A and B, there were no significant differences between the results obtained from the three groups at third and sixth month.

The OCN levels were reduced at the sixth month for all subjects (Fig. 2A and Table 2); however, in our previous study, HYP observed an increase in proliferation of osteoblast cells in vitro examination (Kim et al. 2011). This may be attributed to the formation of OCN in both bone formation and resorption. It has been known that OCN is used as a formation marker; however, it also reflects bone resorption, as during bone resorption it is degraded and up to 70% enters circulation (Delma 1988). Because OCN in circulation may be both newly synthesized during bone formation and released during resorption, there is some question whether OCN should be considered a marker of bone formation or resorption processes (Christenson 1997), besides, OCN has a circadian rhythm in peaking at approximately 0400, so care is needed for consistency in the time of sampling which is practically difficult (Delma 1988; Brown et al. 2009).

**Table 2:** Mean values ± SEM of bone formation markers at 0, 3, and 6 months (M)

	OCN (ng/mL)			BAP (*μ*g/L)		
		
	0 M	3 M	6 M	0 M	3 M	6 M
WA-1	4.39 ± 0.22	4.48 ± 0.23	3.95 ± 0.19	9.30 ± 0.62	9.83 ± 0.63	9.88 ± 0.64
WA-2	4.94 ± 0.28	4.87 ± 0.30	4.17 ± 0.22	10.64 ± 0.82	11.14 ± 0.85	10.41 ± 0.82
WA-3	4.65 ± 0.26	4.65 ± 0.22	4.17 ± 0.24	9.60 ± 0.52	10.72 ± 0.66	9.66 ± 0.52

SEM, standard error of mean; OCN, osteocalcin; BAP, bone-specific alkaline phosphatase.

BAP is produced in extremely high amounts during the bone formation and is, therefore, an excellent marker of bone formation activity (Christenson 1997). Moreover, the advantages of using BAP in clinical practice include sample stability, low biological variability, lack of renal function concerns, and very low diurnal variability (Rosen et al. 1997). As shown in Figure 2B and Table 2, subjects receiving HYP (WA-3 group) showed a trend of increasing BAP levels, compared to other subjects at third month of examination, however, unexpectedly at sixth month BAP gave reduced levels compared to WA-1 group. However, there were no significant differences in BAP levels among all groups.

Although HYP stimulates osteoblast cell proliferation as previously reported (Leem et al. 2004; Kim et al. 2008, 2011), it shall be difficult to expect a marked increase in bone formation markers of grown adults, especially the perimenopausal women.

### Bone resorption markers

**Figure 3:**
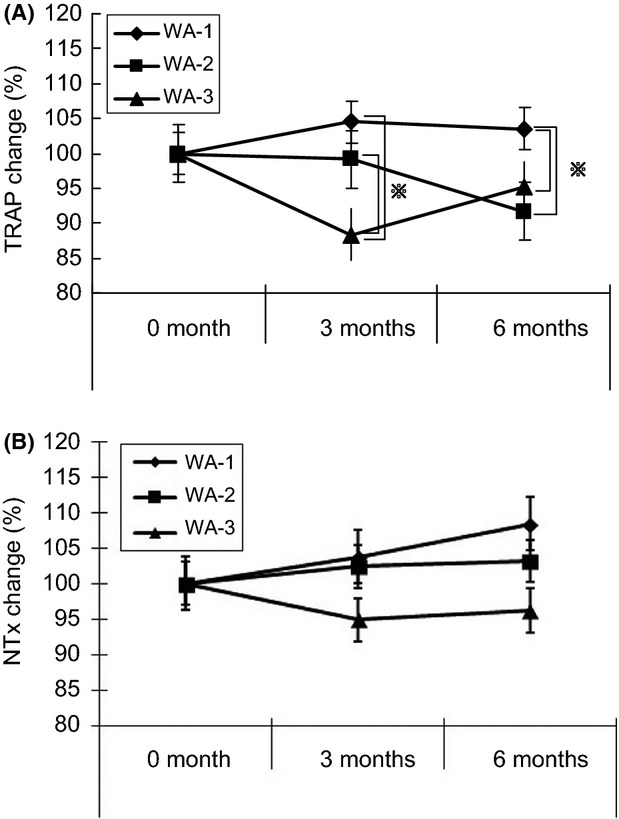
Tartarate-resistant acid phosphatase (TRAP) change rate (A) and *N*-telopeptide (NTx) change rate (B) in women (mean ± SEM) in WA-1, WA-2, and WA-3 groups after 0, 3, and 6 months.

On the other hand, as shown in Figure 3A and Table 3, the bone resorption marker TRAP showed significantly decreased levels at the third month in WA-3 group compared to other two groups (*P* < 0.05). At the sixth month, there were also significant differences in TRAP levels in WA-3 and WA-2 groups compared to WA-1 group (*P* < 0.05). Bone resorption markers reflect osteoclast activity and/or collagen degradation. TRAP is an enzyme expressed when differentiating to osteoclast cells and also a sole direct-indicator of osteoclast numbers and bone resorption activity. So, TRAP is a bone resorption marker that was reported to be increased in patients with osteoporosis (Hochberg et al. 2002). The results of bone resorption markers suggest that HYP intake would have an antiosteoporotic effect that leads to better bone metabolism.

**Table 3:** Mean values ± SEM of bone resorption markers at 0, 3, and 6 months (M)

	TRAP (mU/dL)			NTx (nmol BCE/L)		
		
	0 M	3 M	6 M	0 M	3 M	6 M
WA-1	253.76 ± 22	265.33 ± 32	262.71 ± 34	10.10 ± 0.52	10.48 ± 0.52	10.95 ± 0.52
WA-2	294.60 ± 33	292.00 ± 44	270.05 ± 33	11.69 ± 0.52	11.97 ± 0.52	12.06 ± 0.52
WA-3	247.09 ± 34	218.32 ± 54	235.36 ± 44	12.46 ± 0.52	11.82 ± 0.52	11.98 ± 0.52

SEM, standard error of mean; TRAP, tartarate-resistant acid phosphatase; NTx, N-terminal collagen telopeptide; BCE, bone collagen equivalent.

As shown in Figure 3B, and Table 3, only WA-3 group showed a decreasing trend of NTx level compared to the other two groups, however, there were no significant differences observed in NTx levels among all groups. The bone resorption marker NTx is considered as one of the most widely used in clinical practice and there are studies suggesting that NTx excretion is a significant predictor of fracture risk in postmenopausal women (Delma 1988; Christenson 1997; Garnero et al. 2000).

It was noticed that formation and resorption markers' levels showed much significant change at third than at sixth month examination. In agreement with these results, it has been reported that clinical trials of various antiresorptive therapies have a rapid decrease in bone resorption markers within few weeks after initiation of therapy, and then plateauing after 6 months (Cremers and Garnero 2006; Garnero 2008).

### Bone mineral density

**Figure 4:**
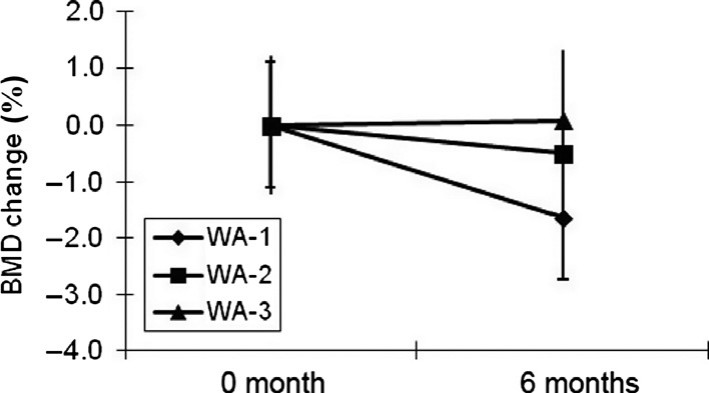
Bone mineral density (BMD) change rate (percent change from baseline; mean ± SEM) in women in WA-1, WA-2, and WA-3 groups after 0 and 6 months.

Moreover, WA-3 group showed inhibition in the loss of bone density and BMD of lumbar spines maintained healthy numerical values, while values in WA-1 and WA-2 groups showed reduction in BMD values at sixth month (Fig. 4). The measurement of BMD is the most important tool for monitoring response to therapy in osteoporotic patients. This technology provides a sensitive means for diagnosing decreased bone mass and predicting fracture. Measurement of BMD using dual energy X-ray absorptometry (DEXA) is perhaps most useful, having an accuracy exceeding 95%, especially for the femoral and lumbar spines. Thus, BMD is considered the gold standard for diagnosis and treatment monitoring and, therefore, used for most clinical studies (Elliott and Binkley 2004). In this study, HYP exhibited a slight increase in BMD compared to both baseline and other groups at 6 months. It has been reported that after initiation of antiresorptive therapy, either stability or an increase in bone mass is considered as a positive response, in that stable BMD is associated with reduced fracture risk (Hochberg et al. 2002). However, using BMD to determine a response to therapy may take 1–2 years. Therefore, BMD findings for HYP intake in this study will need more investigations in a large-scale human trial for a longer period.

Recent studies have provided evidence that egg-derived bioactive peptides play a vital role in human health and nutrition. These evidences suggest that, due to valuable biological functions with health beneficial effects, egg-derived bioactive peptides have potential as active ingredients for preparation of various functional foods or nutraceutical and pharmaceutical products (Anton et al. 2006). HYP has been reported to have antiosteoporotic effects that have been confirmed both in vitro and in vivo. In this study, it is suggested that HYP could be a potential food origin candidate that would have antiosteoporotic effect especially for perimenopausal women. The combination of vitamins, collagen, and isoflavone tended to improve the bone metabolism by suppression of bone absorption (Guillerminet et al. 2010; Taku et al. 2010; Levis and Lagari 2012). Addition of HYP enhanced the beneficial activity of the mixture. Moreover, it has been reported that bioactive peptides produced by enzymatic hydrolysis method is preferred, especially in the food industries because of lack of residual organic solvents or toxic chemicals in the products (Harnedy and FitzGerald 2012). Nevertheless, the HYP ingested for this clinical test is a mixture of peptides, and although the biologically active substance and its mechanism are still being investigated, it is of a great interest that we verified the physiological function from the food with the great familiarity in our daily lives.

We could conclude that supplementation with 100 mg HYP daily has antiresorptive and antifracture effects in perimenopausal women that have been confirmed by bone markers and BMD values. Besides, according to the result of biochemical examination, there were no abnormal findings or side effects reported during the 6-month study. Hence, oral intake of 100 mg HYP daily for 6 months is considered to be safe.
